# Supplementary material for: Cell size reduction scales spindle elongation but not chromosome segregation in C. elegans
Source: Res Sq. 2025 Dec 1:rs.3.rs-7923379. Preprint. [Version 1] doi: 10.21203/rs.3.rs-7923379/v1 (PMC12687828; doi:10.21203/rs.3.rs-7923379/v1)
Supplement: Supplement 1 [file NIHPPrs7923379v1-supplement-1.pdf]

Supplementary Files

This is a list of supplementary files associated with this preprint. Click to download.

- ManuscriptFiguresupplementary.pdf
- Movie1.mov
- Movie2.mov
- Movie3.mov
- Movie4.mov
- Movie5.mov
